# Breast Cancer Attributable Costs in Germany: A Top-Down Approach Based on Sickness Funds Data

**DOI:** 10.1371/journal.pone.0051312

**Published:** 2012-12-10

**Authors:** Emil Victor Gruber, Stephanie Stock, Björn Stollenwerk

**Affiliations:** 1 Institute of Health Economics and Health Care Management, Helmholtz Zentrum München, Neuherberg, Germany; 2 Institute of Health Economics and Clinical Epidemiology, University of Cologne, Cologne, Germany; Academic Medical Center, The Netherlands

## Abstract

**Background:**

Breast cancer is the leading cause of death from cancer among women in Germany. Despite its clinical and economic relevance, no attributable costs for breast cancer have been reported for Germany so far. The objective of this study is to estimate age-specific breast cancer attributable health expenditures for Germany.

**Methods:**

Sickness fund data from 1999 representing about 26 million insured (i.e. 32% of the total German population) have been analysed using generalized additive models and the error propagation law. Costs have been inflated to 2010.

**Results:**

Breast cancer attributable costs decreased with age. Among breast cancer patients aged 30–45 years, about 90% of all health expenditures were due to breast cancer, whereas in breast cancer patients aged 80–90 years, about 50% were due to breast cancer. Breast cancer attributable costs amounted to about €9,000 annually for patients below 55 years of age and declined to about €3,000 in 90-year-old breast cancer patients.

**Conclusion:**

This analysis provides estimates of attributable breast cancer costs in Germany. Compared with the international literature, the estimates were plausible but had a tendency to underestimate breast cancer attributable costs.

## Introduction

Breast cancer is one of the most frequently occurring forms of cancer [1], [2]. Breast cancer ranks first with the highest prevalence of all cancer types, accounting for 17.9% of all cancer cases [2]. In 2004, about 370,100 new cases were registered in Europe, accounting for 12.8% of all sites except non-melanoma skin cancer [1]. In Germany, 57,000 new cases of breast cancer are registered each year. They account for 27.8% of all cancer cases in German women [3]. Additionally, breast cancer is the main cause of cancer-related deaths in women worldwide [4], [5]. In Europe, the mortality rate reached 26.0 per 100,000 in 2006 with Germany being above this average figure at 26.5 per 100,000 [4]. The economic impact of breast cancer is high. The breast cancer attributable lifetime costs, based on United States (US) Medicare data, ranged from $37,306 for women diagnosed at 65–69 years of age to $19,493 for women aged 85 years and older at diagnosis [6].

There are numerous possible interventions to prevent, detect or treat breast cancer [7]. Probably the most important screening tool is mammography screening in women aged 55–75 years [8]. Numerous treatment methods exist for breast cancer such as surgical removal of the tumour and, depending on the stage, a combination of radiotherapy, chemotherapy, hormonal therapy and immunomodulation [7]. For the prevention of breast cancer, a series of risk factors have been reported, such as overweight in post-menopausal women or the use of exogenous sex steroid hormones [7].

The need to evaluate such interventions is accompanied by numerous publications in this field [9], [10], [11], [12]. Such evaluations are useful for the deployment of resources such as manpower, facilities, equipment and knowledge more efficiently. Furthermore, they help to set guidelines for future studies [13], [14]. In order to perform economic evaluations, cost data are needed. Although some studies estimate the overall costs of patients with breast cancer, the economically relevant costs are those that may be avoided by the intervention.

Even though some economic analyses regarding breast cancer exist [15], [16], [17], breast cancer attributable costs have not yet been reported for Germany. The objective of this study is to estimate breast cancer attributable health expenditures for Germany.

## Methods

### Data

The data used for this analysis refer to the year 1999 and were collected to support an experts’ report to prepare a German health care reform programme [18], [19], [20]. Data from all insurants in six major German sickness funds were included. All six sickness funds reported data with respect to the prevalence of seven chronic diseases (diabetes mellitus, coronary artery disease (CAD), hypertension, asthma, heart failure (HF), breast cancer and stroke). The disease status was measured using ICD-9 coding (International Statistical Classification of Diseases and Related Health Problems) and the Anatomical Therapeutic Chemical Classification System (ATC) code (Table 1). Cost data were based on four of the six sickness funds (i.e. hospital costs, medication spending and sickness benefit). Hospital costs reported include all costs for inpatient care, i.e. physician costs, medication costs, general costs for hospital stay and nursing care. However, outpatient care costs were not included.

**Table 1:** The diseases featured in the dataset and their coding.

Disease	ICD-9 code	ATC code
Asthma	493	RO3^a^
Diabetes mellitus	250	A1O^a^, VO4CA^a^
Hypertension	401–405	CO2^a^, CO3^a^, CO7^a^, CO8^a^and CO9^a^
Heart failure	428	CO1AA, CO1AB, CO3^a^and CO9^a^
Coronary artery disease	410–414	CO1D
Stroke	430–438	
Breast cancer	174	

^a^The original study comprised seven chronic diseases and analysed the prevalence and costs of the chronic disease of interest among beneficiaries of the German SHI based on administrative claims data.

For this analysis, two highly aggregated datasets were supplied [19], [20], [21]. In both datasets, each row represents all subjects with the same characteristics with respect to age, gender and comorbidities. Besides the patient characteristics, a further column reports the observed number of days of insurance (i.e. the total number of days that the women – aggregated by age and comorbidities – were insured in 1999). The first dataset was based on all six sickness funds. It corresponds to 26 million insurants (15.6 million women) and was assumed to represent the population of Germany appropriately (32% of the German population, 37% of the German female population). The second dataset also includes a column for the average health expenditures per day of insurance. This dataset corresponds to all 14 million insured (8.5 million women) in the four cost reporting sickness funds (10% of the German population, 20% of the German female population). All costs were converted to euros (€; exchange rate 1.95583:1) and inflated to 2010.

### Primary Target Variables

The primary target variables of this study were, first, the absolute health expenditures of women with breast cancer, second, the costs attributable to breast cancer, and third, the share of costs in breast cancer prevalent subjects that is attributable to breast cancer.

### Regression Model for Cost Prediction

Based on the above described dataset of the four sickness funds, a generalized additive model (GAM) [22] was fitted to predict costs for given patient characteristics. As breast cancer in women accounts for over 99% of the total breast cancer incidence, only women were included in the analyses [6], [23].

GAMs are an extension of the generalized linear model (GLM) that allow for a non-linear influence of covariates [22]. The response variable in the regression model was the costs per day of insurance. Costs were assumed to be Gamma distributed and a log-link function was applied [21], [22]. The days of insurance were used as the weight. Besides age and comorbidities, pairwise interactions of these were also allowed as covariates. Comorbidities and interactions among these were represented as binary variables (1 = prevalent subjects, 0 = non-prevalent subjects). Age and all interactions with age were modelled as smooth terms. Thin plate regression splines were used for smoothing [22]. A backward variable selection was applied including all variables that were significant at the 5% level [21]. If an interaction term was included, the main effect was also included even if it was not significant.

### Further Calculations

The fitted regression model was used to perform several cost predictions. First, costs were predicted for all women with breast cancer based on the dataset of all six sickness funds. Second, we reused the patient characteristics of the first cost prediction but set the breast cancer status to ‘false’. In other words, we designed a hypothetical non-prevalent (breast cancer) cohort that corresponds to those with breast cancer in terms of comorbidities.

The first cost prediction was used to calculate age-specific estimates of average health expenditures for women with breast cancer. Furthermore, age-specific breast cancer attributable costs were calculated. These resulted from the difference in the averaged costs of both cost predictions. As well as this absolute cost difference, we also calculated the share of health expenditures that can be avoided by preventing breast cancer. This share results from 1 minus the quotient of the average value of the second cost prediction (breast cancer status set to ‘false’) by the average value of the first cost prediction (women with breast cancer).

To assess the stochastic uncertainty of our target variables (i.e. average costs, attributable costs, share of avoidable costs), we applied Gauss’s error propagation law [21]. This is an analytical approach, where the variance of the target variable is calculated based on the variances of the underlying input variables (here: individual cost predictions, days of insurance). Details of this approach have already been published elsewhere [21].

Statistical analysis was performed using the software R (version 2.11.0) [24], and GAMs were fitted with the R library ‘mgcv’ [25]. Derivations to apply the error propagation law were calculated using the software Mathematica (version 7).

## Results

The regression model that was used to predict health expenditures for given patient characteristics is displayed in Table 2. Based on highly aggregated data that represent more than 14 million insured, the adjusted R^2^ was reasonably high (0.988). Within backward selection, the coefficients of the interactions of ‘CAD and HF’, ‘asthma and HF’, ‘diabetes and CAD’ and ‘diabetes and HF’ were removed.

**Table 2:** The estimates, standard errors and test statistics of the variables in the generalized additive model.

	Parameter estimate	Standard error	T statistic	P value
(Intercept)	–0.38	0.33	–1.15	0.25
Diabetes	1.74	0.31	5.56	<0.001
CAD	–0.62	0.53	–1.17	0.24267
Hypertension	1.91	0.16	11.81	<0.001
Asthma	1.26	0.06	20.62	<0.001
HF	2.03	0.20	10.34	<0.001
Breast cancer	2.26	0.76	2.98	0.003
Stroke	2.38	0.13	17.81	<0.001
Diabetes and hypertension	–0.42	0.02	–22.35	<0.001
Diabetes and asthma	–0.12	0.02	–6.52	<0.001
Diabetes and breast cancer	–0.48	0.04	–11.66	<0.001
Diabetes and stroke	–0.24	0.02	–10.90	<0.001
CAD and hypertension	–0.12	0.02	–5.81	<0.001
CAD and asthma	–0.08	0.02	–5.07	<0.001
CAD and breast cancer	–0.36	0.04	–9.25	<0.001
CAD and stroke	–0.26	0.02	–12.54	<0.001
Hypertension and asthma	–0.33	0.02	–20.85	<0.001
Hypertension and HF	–0.11	0.03	–4.07	<0.001
Hypertension and breast cancer	–0.41	0.04	–11.60	<0.001
Hypertension and stroke	–0.42	0.03	–13.38	<0.001
Asthma and breast cancer	–0.34	0.04	–9.41	<0.001
Asthma and stroke	–0.23	0.03	–8.55	<0.001
HF and breast cancer	–0.15	0.04	–4.03	<0.001
HF and stroke	–0.25	0.03	–8.59	<0.001
Breast cancer and stroke	–0.87	0.06	–13.80	<0.001
**Approximate significance of smooth terms**				
	**Estimated degrees of freedom**	**Estimated** **rank**	**F statistic**	**P value**
Age	9.0	9	855	<0.001
Age*diabetes	8.9	9	135	<0.001
Age*CAD	6.8	7	52	<0.001
Age*hypertension	4.8	6	72	<0.001
Age*asthma	8.9	9	12	<0.001
Age*heart failure	8.4	9	14	<0.001
Age*breast cancer	5.8	7	80	<0.001
Age*stroke	2.4	3	85	<0.001

HF, heart failure; CAD, coronary artery disease.

**Figure 1:**
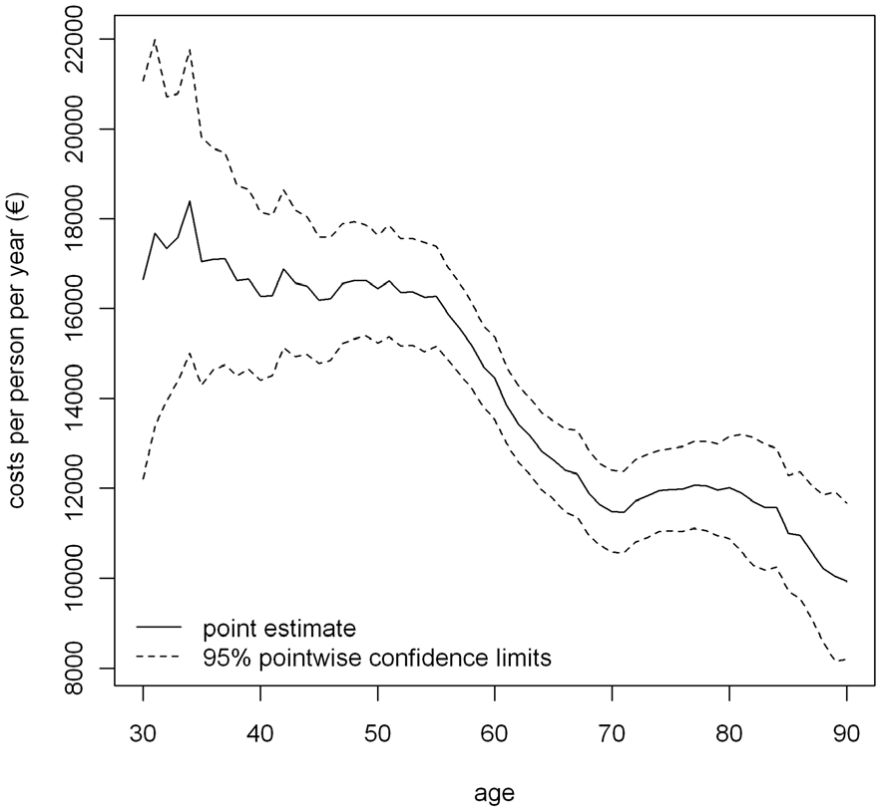
The absolute health expenditures of women with breast cancer (costs in 2010 euros).

The age-specific health expenditures in women with breast cancer, expressed as annual costs per woman, are strongly associated with age (Figure 1). Although the costs are about €10,000 in 30- to 55-year-old women, costs decrease to approximately €7,000 for 70-year-old women and increase to €7,500 for 80-year-old women. Costs are lowest in 90-year-old women reaching just above €6,000. The variance of the absolute health expenditure in women less than 55 years of age is significantly higher than in women above 55 years. For 30-year-old women, for example, the standard error (SE) of the absolute health expenditures is equal to €1,390, whereas it is €285 for 70-year-old women with breast cancer.

**Figure 2:**
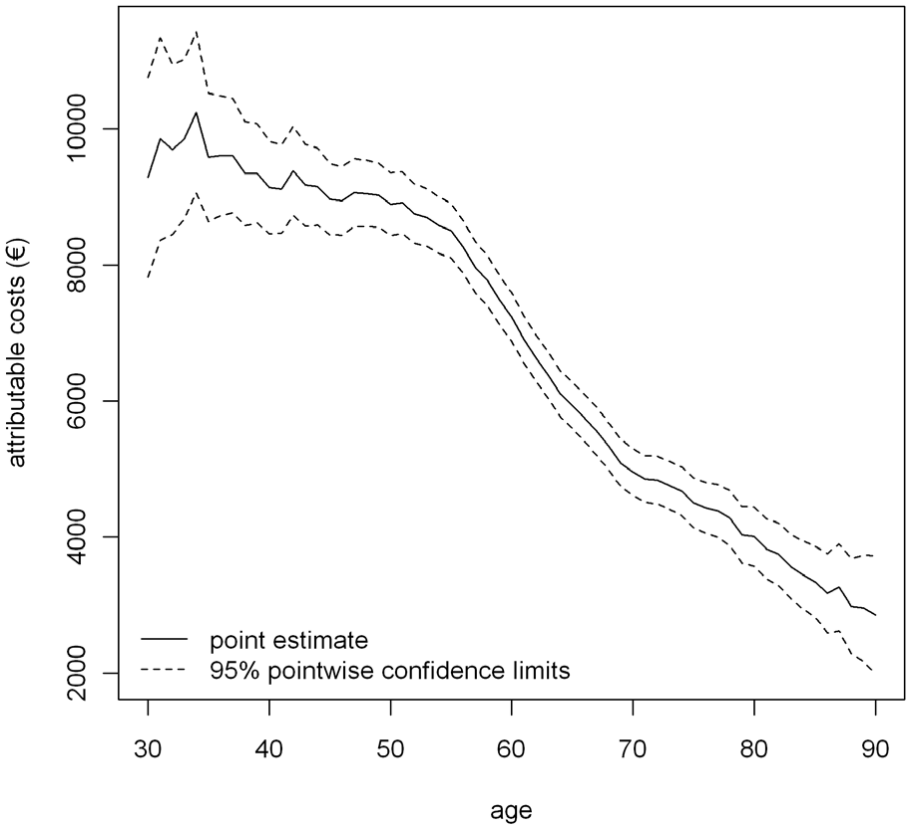
Breast cancer attributable costs (in 2010 euros).

The breast cancer attributable costs are also negatively associated with age (Figure 2). The attributable costs describe a curve from about €9,000 for 30- to 55-year-old women to about €8,500 in 57-year-old women, then decrease rapidly to about €5,000 for 69-year-old women and continue decreasing to less than €3,000 for 90-year-old women. Variance is highest in women below 45 years of age (standard error (SE) = €353 for age 40 years) and lowest between 55 and 70 years of age (SE = €157 for 60-year-old women).

**Figure 3:**
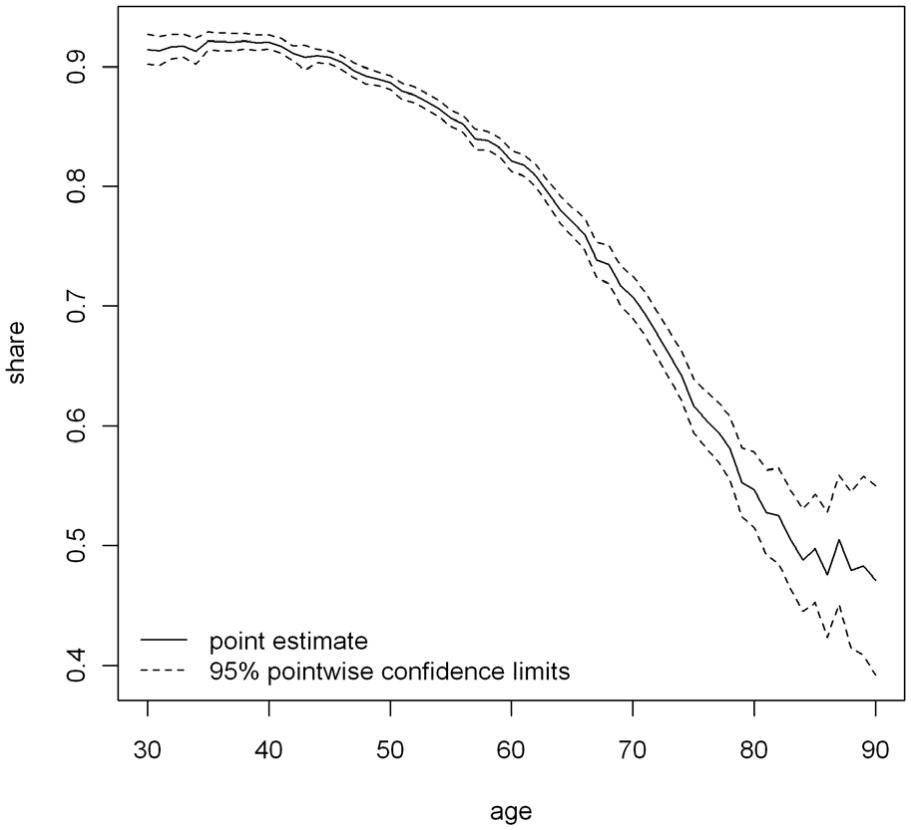
The share of breast cancer attributable health expenditures.

The share of breast cancer attributable health expenditures is negatively associated with age (Figure 3). Although about 90% of the health expenditures can be avoided in 30-year-old women with breast cancer, this amount decreases to 70% for 70-year-old women and about 47% for 90-year-old women. In women aged 30–75 years, the estimates are relatively precise with respect to stochastic uncertainty (SE = 0.7% for 30-year-old women and 0.3% for 50-year-old women). In subjects aged 75 years and older, the corresponding confidence limits are much wider (SE = 3% for 85-year-old women and 4.1% for 90-year-old women).

## Discussion

In this study, we estimated the attributable health expenditures of breast cancer in Germany using sickness fund data. This has been the first time that estimates of breast cancer attributable health expenditures have been provided for Germany. One specific characteristic of this study is that it is based upon a large sample size that was collected within an experts’ report commissioned to extend the risk adjustment scheme within the German statutory health insurance [18], [20], [26].

We found that the share of preventable costs decreased strongly with age. This is because comorbidities are much more common among older patients than in younger ones [20]. If comorbidities are low, as in most of the younger women, the highest share of health expenditures results from breast cancer. If breast cancer could be prevented in these women, a large share of the costs would be avoided. At more advanced ages, where comorbidities are much more common, breast cancer costs amount to a smaller part of overall health expenditures.

However, not only does the share of health expenditures attributable to breast cancer decrease with age, but the absolute attributable breast cancer costs also decline. This has been observed previously: in hospitalized women in California with a primary diagnosis of ‘breast cancer’, mean costs decreased with age, ranging from $7,510 in women younger than 35 years to $4,688 for women aged 75 years and above [6]. It is unclear whether this is because elderly women request less aggressive treatment or if treatment options are withheld at older ages. Furthermore, in our study, costs included sickness benefit, which is only paid to salaried employees; this also leads to a decline in costs with age.

When comparing our findings with other European data, the estimates appeared very plausible. Whereas we estimated annual costs of about €10,000 for women in their thirties and about €5,000 in 70-year-old women, a study conducted in Belgium reported total direct costs of women with breast cancer of €10,071 in the year of diagnosis, with a steep decline to €3,245 in the second year [27]. In the US, however, breast cancer attributable costs appear to be significantly higher. A study analysing Medicaid fee-for-service programme data found that the costs of women with breast cancer amount to $16,345 annually [28]. The study used administrative claims data and had a 1-year follow-up [28]. Although these values are relatively close to our findings, a study also analysing Medicaid data showed different results: for patients with local breast cancer, they reported costs of $14,341 after the initial 6-month period and $22,343 after 2 years. For patients with metastasized breast cancer, however, they reported costs of $117,033 after 2 years of follow-up. The results that differed most from our study are US estimates collected within a bottom-up approach: annual total costs of subjects with breast cancer were reported as $75,190 [29]. Although no breast cancer attributable costs for Germany have been reported yet, the costs of febrile neutropenia/leukopenia chemotherapy have been published recently. They amount to €1,900 per episode [30].

There are several limitations to this study: first, some cost categories (i.e. outpatient care costs) were not included. In contrast, sickness benefit was included, even though sickness benefit in general is considered as transfer costs and does not belong to the direct costs. It would thus be valuable to report costs stratified by cost category; however, the aggregated data available for this analysis did not allow for stratification.

Second, regarding the covariates used for adjustment, our choices were limited because of data availability. Adjustment variables have to be chosen carefully in order not to bias the results. We assumed that the variables used for adjustment were not affected by breast cancer. Although this assumption appears reasonable, important cost factors confounded with breast cancer prevalence might potentially be missing.

Third, in this study, the prevalence of breast cancer was defined using ICD-9 coding. For breast cancer, this coding is limited to malignant neoplasm of the female breast (code 174), giving no information about the cancer stage. Other definitions are possible. For example, Barron et al. used more categories, which also included ‘carcinoma in situ of the breast’ (ICD-9 code 233.0), ‘neoplasm of uncertain behaviour of the breast’ (238.3) and ‘neoplasm of unspecified nature of the breast’ (239.3) [31].

Finally, the data used for this analysis are from 1999, and much has changed in the last decade in breast cancer detection and treatment. While it may be worthwhile to use risk adjustment data for more current analyses, breast cancer has not been included in the German risk adjustment scheme.

The perspective of cost measurement is known to greatly influence the results [32], [33]. This analysis was performed from the statutory health insurance perspective, and thus indirect costs have not been included. However, previous studies have shown that indirect costs account for 70–85% of the total costs. In particular, the indirect costs of cancer were substantial for the German economy [34]. Future studies should calculate health care expenditures based on a societal perspective (including out-of-pocket spending, productivity loss and intangible costs, i.e. loss of quality of life or loss of years lived).

In conclusion, we have delivered the first breast cancer attributable cost estimates for Germany. Considering other breast cancer cost studies, our estimates appear to have a tendency to underestimate attributable breast cancer costs. The methodological approach appeared to be reasonable and seems to be a valid tool for future analyses.
